# A Text Structuring Method for Chinese Medical Text Based on Temporal Information

**DOI:** 10.3390/ijerph15030402

**Published:** 2018-02-27

**Authors:** Runtong Zhang, Fuzhi Chu, Donghua Chen, Xiaopu Shang

**Affiliations:** Department of Information Management, Beijing Jiaotong University, Beijing 100044, China; rtzhang@bjtu.edu.cn (R.Z.); october_chu@163.com (F.C.); chendonghua@bjtu.edu.cn (D.C.)

**Keywords:** electronic medical records, temporal information, text structuring method, Chinese, information entropy

## Abstract

Chinese Electronic Medical Records (EMRs) contains a large number of complex medical free text which includes a variety of information, such as temporal information, patients’ symptoms and laboratory data. However, as an important knowledge base, these unstructured text data in EMR are hard to process directly by computer to support further medical research. This paper proposes a novel text structuring method to extract knowledge from EMR texts and reorganize them in chronological order according to the temporal information in the text. By implementing some entropy-based algorithms as contrast, experiments evaluate the performance of the proposed method, which indicates the new method can significantly reduce the complexity of EMR text. This work is significant in structuring the EMR free text into temporal-structured data for further medical analysis.

## 1. Introduction

An Electronic Medical Record (EMR) is a professional document that contains all data generated during the treatment process. The EMR can include data of various formats, such as numerical data, text, and images. In China, quick development of hospital information technologies has brought a rapid accumulation of medical data, and a huge amount of these data are in the form of unstructured text, leading to difficulties in medical information management in the era of big data [1]. The standardization of Chinese EMRs has been implemented successfully in many hospitals, and it helps enable the management and analysis of medical records in different ways. However, many medical researchers are submerged in huge amounts of clinical data in their studies, because it is hard for them to discover the hidden knowledge laying in the massive amount of medical text, which is a great challenge to efficiently use the text in EMRs in the medical field [2]. The temporal information, recorded in natural language, is an important clue in EMR text related with the chief complaint, history of present illness, past history of illness and so on. In this study, based on the temporal information from the EMR text, we propose a Medical Text Reconstruction method based on Temporal Information (MTRTI) to establish the mapping relationship between medical concepts and clinical facts, which is temporal-structured. It helps medical researchers utilize the EMR text in an easier way to investigate the clinical knowledge that lies in the text. Besides, the processed text data can be further used easily by computer-aid analysis according to the specific needs [3]. 

The remainder of the paper is organized as follows: Section 2 reviews related work. Section 3 describes the framework of MTRTI and the modules in it. Section 4 evaluates the performance of MTRTI based on information entropy theory. Then, conclusions are given in the last section.

## 2. Related Work

Mining the knowledge in medical texts, such as EMR text, has been attracting more and more attention, as people have noticed that the text data is as important as other structured data which contains a lot of information and knowledge. However, it is difficult to directly analyze the medical data by traditional computer algorithms without pre-processing [4]. As one of the characteristics of medical text, all EMR texts contain temporal information, which describes the disease progression and clinical process. Although mining the knowledge lies in the EMR text is a challenging work [5,6,7,8,9], extracting the temporal information of medical events is the basis for further clinical analysis. Reeves et al. [10] divided the temporal information into four aspects that are namely date, time, duration of time and fixed time by recognizing and analyzing time reference expressions. In order to extract the time relationships effectively, Norén et al. [11] proposed a time measure approach for time management, including the observation/expectation rate after a two-year control period. Based on the pattern discovery methods of the two million patient data samples in the United Kingdom, this was proved practical. Towards the studies of the relationship between temporal information, considering additional information will help explain more medical facts. Hanauer et al. [12] proposed an approach to extract timestamp-based ICD-9 coding from 16 million patients’ data. In order to study the classification of temporal expressions from medical free texts, Zhou et al. [13] established a time-constrained model to achieve reasoning ability based on temporal information, which successfully extracted all kinds of temporal information from the abstracts of hospital discharges.

Temporal information in EMR is essential in mining the medical pattern and normal in clinical research, Zhang et al. [14] used the health information systems at a mental hospital to investigate drug reactions and identify the adverse events which were later associated to the specified medicine and dose. Aiming at irregular and multi-dimension data, Singh et al. [15] proposed three kinds of methods to establish prediction models using temporal health data, which has a good prediction ability in studies of renal function loss. Xu et al. [16] put forward a framework based on patient information to translate Chinese medical text into a triple “time, event, descriptions” structure, the output of which could be the explanation or the consequence of medical events. For the studies of time changing trends of personal healthcare archives, building the relationship between medical events and temporal information helps in depth study of the use of temporal information from EMR texts [17]. 

These above works indicate that an effective structuring method to analyze medical free text is important in mining the latent medical knowledge. Some of them introduce SNOMED CT and ICD-10 into the structuring process, which improves the standardization of the data. However, these researches are done with the idea of extracting information from medical texts, rather than reconstructing them into a structured data format. Extracting data from text is an important way to structure the text data. However, some information from data may be missed during the extraction process. In order to process the Chinese EMR text into template-structured data, reduce the semantic uncertainty caused by unstructured medical text, and avoid missing data when extracting data from text, this paper proposes a new method called MTRTI to reconstruct the EMR text into a computer friendly data format. Text data reconstruction, which is able to keep all of the information in the original text, is different from the idea of extracting data from text. The details of the new method are described in the following sections.

## 3. Framework and Details of MTRTI

### 3.1. The Framework of MTRTI

**Figure 1:**
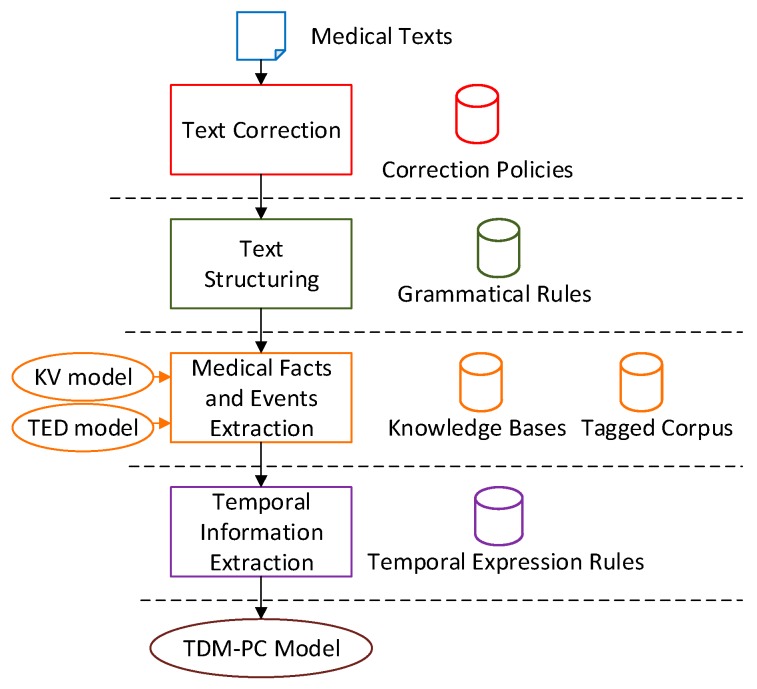
The architecture of MTRTI.

MTRTI includes several modules, and Figure 1 describes its framework. Generally, these modules are Text Correction module (TC), Text Structuring module (TS), Medical Facts and Events Extraction module (MFEE), and Temporal Information Extraction module (TIE). Each module needs the support of a corresponding rule base or database. The TC corrects the spelling mistakes based on correction policies. The TS splits the texts according to grammatical rules. Based on the medical domain knowledge bases and tagged corpus, the KV model and TED model work to describe the medical facts and events in the MFEE. The TIE processes the temporal expressions and establishes the Temporal Development Model in Patient Condition (TDM-PC) model for the text.

### 3.2. Text Correction Module

In this framework, the functions of some basic modules can be implemented by existing technology and tools respectively. TC module is the basic text pre-processing stage that enables the input of the text data be well formatted, and correct some typos exist in the text. Correction rules are the core of TC as given in Algorithm 1.
**Algorithm 1.** Text correction algorithm.**INPUT**: *medical_text, map_path* **OUTPUT**: *standardized_medical_text* **VARIABLES** ( **DEF**
*medical_text*: a part of medical texts from electronic medical records **DEF**
*map_path*: the path of a mapping database for text correction rules **DEF**
*standardized_medical_text*: the standardized text returned to the platform **DEF**
*map*: the mapping list read from *map_path* ) *map* = READ_DB(*map_path*) *medical_text* = REMOVE(*medical_text*, EMPTY_STR) *medical_text* = REMOVE(*medical_text*, LINE_WRAP_STR) **FOR**
*i* = 0 **TO** LENGTH(*map*)   *medical_text* = *medical_text*.REPLACE(*map[i]*.original, *map[i]*.replacement) **END FOR** *standardized_medical_text* = *medical_text* **RETURN**
*standardized_medical_text*

The above algorithm is focused on removing misused characters and correcting explicit grammar errors in the raw text. Text correction policies can be defined in a database which stores the mapping rules between error string and correct string. Removing the non-standardized characters in raw medial text helps simplify the analysis process of complicated Chinese medical texts in EMR.

### 3.3. Text Structuring Module

TS is one of the most important modules in the framework, for it concerns the precision of the result. The EMR text contains the description of the patient’s illness, health history, as well as laboratory data, and so on. These texts are recorded by physicians using medical terms, which differ in style from our daily language, being less subjective and much shorter in length. 

For example, the sentence from a clinical note “否认肝炎、结核、疟疾病史，高血压史、冠心病史20年，糖尿病史20年 (Denial of Hepatitis, Tuberculosis and Malaria; 20-year history of Hypertension and Coronary Heart Disease)” is composed by words and short sentences, without prepositions and auxiliary words, and hence very simple. We are able to acquire information from the above texts if we can split the sentence into phrases with correct semantics, such as 否认 (Denial)|肝炎 (Hepatitis)|结核 (Tuberculosis)|疟疾病 (Malaria)|高血压 (Hypertension)|冠心病 (Coronary Heart Disease)|20年 (20 years). Algorithm 2 describes the specific rules of TS.
**Algorithm 2.** Text structuring algorithm.**INPUT**: *data* **OUTPUT**: *mt* **VARIABLES** ( **DEF**
*data*: raw string **DEF**
*xmldata*: xml-formatted string **DEF**
*mt*: Medical Text Object ) *xmldata* = “<MedicalText>” string[] *sentences* = *data*.Split(‘.’) **FOR**
*i* = 0 **TO**
*sentences*.Length  *xmldata* += “<Sentence>”  string[] *segments* = *sentences*[*i*].Split(‘,’)  Sentence *sentence* = new Sentence()  **FOR**
*i1* = 0 **TO**
*segments*.Length    **IF** string.IsNullOrEmpty(*segments*[*i1*].Trim())      **Continue**    **END IF**    *xmldata* += “<Segment>”    *xmldata* += segments[*i1*]    Segment *segment* = new Segment()    *segment*.Text = segments[*i1*]    *sentence*.Segment=ArrayUtils.AddArrayItem<Segment>(*sentence*.Segment, *segment*)   **END FOR**   **FOR**
*j* = 0 **TO**
*times*.Length     *xmldata* += “</Segment>”   **END FOR**   *mt*.Sentence = ArrayUtils.AddArrayItem<Sentence>(*mt*.Sentence, *sentence*)   *xmldata* += “</Sentence>”   **END FOR** **END FOR** *xmldata* += “</MedicalText>” **RETURN** XmlToObject(*xmldata*)

Note that, the use of grammar rules in Algorithm 2 also depends on actual application scenes in specific language. In Chinese, the analysis algorithm can split the raw text into paragraphs, sentences and segments based on basic Chinese grammatical knowledge.

When trying to analyze the patient condition, TS module makes it possible to extract the useful medical terms and their semantic relationships from those narrative texts. Only when we consider those medical texts by means of semantic approaches can we acquire more hidden knowledge for better analysis.

### 3.4. Medical Facts and Events Extraction Module

The style of medical text is different from the text used in our daily life. Therefore, in the MFEE module, two descriptive models, Key-Value (KV) model and Time-Event-Description (TED) model, are proposed to identify the structure of the knowledge in the narrative texts.

The KV model works to quantify or shorten the description of numerical description in the medical texts. Supposing that *D* is one of the laboratory data, equations (1) and (2) are used to depict such laboratory items:

(1) $$KV_{t}(i)={\left(K_{i},V_{i}\right)}_{t},\;1\leq i\leq n$$

(2) $$D=\{KV_{t}(i)|i\in N\}$$

where *K* stands for the property name, *V* stands for property value which may include the units of measurement, *t* stands for the descriptive contents, *i* is the order in data set *D*. For instance, a piece of Chinese clinical notes “2008年3月11日出现咽痛，给予阿奇霉素抗感染无效，在我院进行血常规检查：WBC2.13 × 10^9^/L, Hb102g/L, PLT177 × 10^9^/L/L. (11 March 2008, occur pharyngodynia; azithromycin was not effective against infection; blood routine examination in our hospital: WBC2.13 × 10^9^/L, Hb102g/L, PLT177 × 10^9^/L/L)” can be described as follows: *KV_t_*(1) = (WBC, 2.13 × 10^9^/L), *KV_t_*(2) = (Hb, 102g/L), *KV_t_*(3) = (PLT, 177 × 10^9^/L/L), *t* = 血常规检查 (blood tests). Thus, we obtain *D* = {*KV_t_*(1), *KV_t_*(2), *KV_t_*(3)}, *n* = 3.

TED model describes the relationship among time, event, and description, and can be described by Equations (3) and (4):

(3) $$TED(i)={\left(t_{i},e_{i},d_{i}\right)}_{p}\in s_{k},1\leq i\leq n,\text{\{}d_{i}\text{\}}\subseteq D$$

(4) $$p=\{s_{i}|\{\{TED_{k}|k\in N\}\subseteq s_{i},i\in N\}\}$$

where *t* stands for time node, *e* represents for medical event description, *d* stands for medical description, *p* is the paragraph description and *s* means segments for a sentence, *p* stands for a single paragraph from the texts, and p may contains many text blocks symbolized as *s*, *i* denotes the *i^th^* text block named *s*. Note that, single text block may contain *k* TED models, where *d_i_* stands for the descriptive information *D* in KV model. Here we use the same example above “2008年3月11日，出现咽痛，给予阿奇霉素抗感染无效，在我院进行血常规检查：WBC2.13 × 10^9^/L, Hb102g/L, PLT177 × 10^9^/L/L. (11 March 2008, occur pharyngodynia; azithromycin was not effective against infection; blood routine examination in our hospital: WBC2.13 × 10^9^/L, Hb102g/L, PLT177 × 10^9^/L/L.)”, we can acquire the following information: TED(1) = (2008-3-11, 出现(occurring), 咽痛(sore throat))、TED(2) = (2008-3-11, 给予阿奇霉素抗感染(given azithromycin against infection), 无效(negative))、TED(3) = (2008-3-11, 进行血常规检查(blood routine examination), (KV(1), KV(2), KV(3)), d3 = (KV(1), KV(2), KV(3)).

Compared with traditional methods that focus on completely analyzing the text structure of medical text and reasoning the semantic relationships among terms in raw text, the use of KV and TED model simplifies the analyzing process by predefining possible text structures that exist in medical text. As a result, many meaningful medical values and events are discovered from the text to support the deep use of medical data. In contrast, traditional methods [18,19] emphasize on the very detailed grammatical relationships in texts, most of which are irrelevant to medical data analysis.

Based on the KV model and TED model, we are able to establish the text descriptive model to structure the Chinese medical texts. In this model, we use temporal information to link all corresponding patient information to acquire a timeline-based model.

### 3.5. Temporal Information Extraction Module

TIE module works to identify the time related text information in the medical text. The works and phrases of time is the same with those we use in daily life, so we can use existing Natural Language Processing (NLP) tools, such as Stanford NLP [20], to realize this function.

### 3.6. Temporal Development Model in Patient Condition

TDM-PC in the framework is to illustrate the illness and clinical events in EMR text. Specifically, it can outline the changes of patient condition by identifying the key information from EMR text in corresponding temporal scenario. It is the final stage to acquire structured data. According to the structure of EMR text and the treatment process, TDM-PC is composed of the following stages: 

Stage 1: Healthy Status

In our proposed model, this is the very beginning part. In this stage, we need to extract the symptom and preliminary diagnosis information from the EMR text. Current patient condition at this time point will be the initial input of patient state for the following stages.

Stage 2: Hospital Diagnosis

First deterioration of the patients’ condition leads to the complete diagnosis of the patient in the hospital. In the hospital, all of the symptoms of patient are recorded in the clinical notes, including physiological and laboratory results. 

Stage 3: Treatment Process

When a patient is admitted, his/her illness related data are recorded continuously during the stay, including the diagnosis, the discussion and decision on the treatment. This stage generates many medical texts.

Stage 4: Medical Treatment

The EMR texts in this stage includes all of the medication information, laboratory data, observation data, etc.

Stage 5: Clinical Follow-Up

When a patient leaves hospital, clinical follow-up information may be acquired according to the health status of patient, to evaluate the long-term changes in the treatment effect of the hospital.

Stage 6: Return Visit

**Figure 2:**
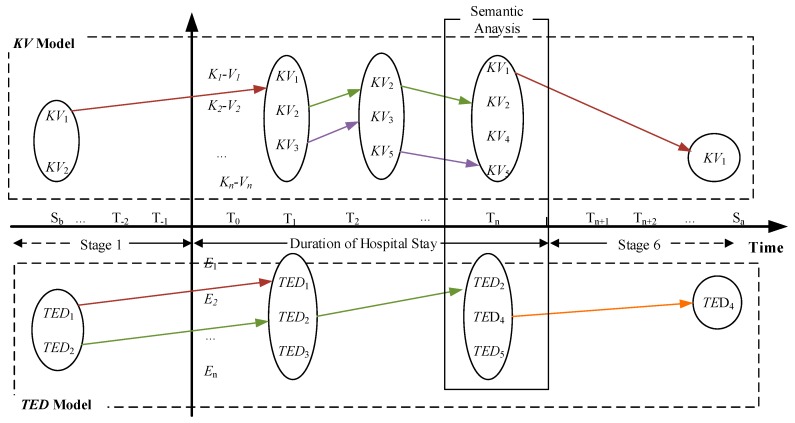
Overview of TDM-PC.

Some of the patients need to come back to hospital for the same illness, which we call a return visit. A return visit is a complete visiting process including all the stages above. These stages cover the whole illness progression of an inpatient from the dimension of temporal sequence. In this study, we use the temporal features to link that information together. In all stages, the condition changes will be recorded in the electronic medical records. Figure 2 illustrates the conceptual model of TDM-PC.

In Figure 2, the horizontal axis is the timeline and vertical axis indicates specific features of the illness description. In EMR text, the different statuses of an illness could be associated with corresponding temporal information in the text. They can be integrated into the different stages of the model.

### 3.7. Data Flow of MTRTI

**Figure 3:**
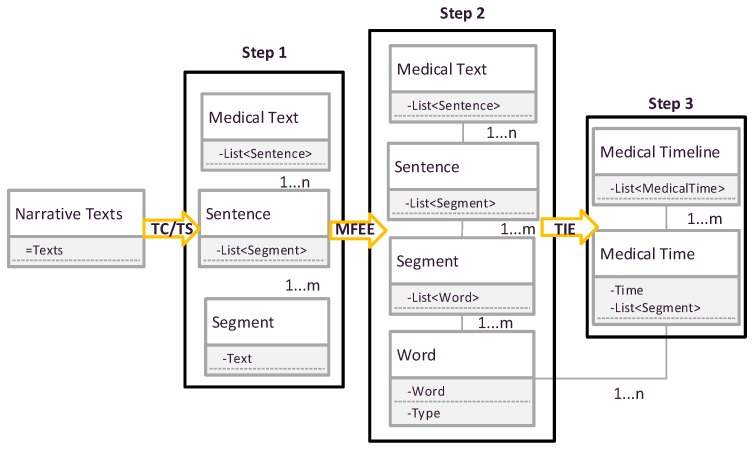
Data flow and data format in the MTRTI.

The changes of the data structure of the processed EMR text in these modules are illustrated as in Figure 3.

In Figure 3, at first, clinical notes are processed by the grammar module that splits the text into paragraphs, sentences and text segments (Step 1). Then, combining with the TS module which labels the part-of-speech for each text segment to generate a phrase lists (Step 2). Next, according to the part of speech of phrase (Step 3), it establishes the MFEE module, which shows the medical facts and events in different time nodes.

## 4. Test on the MTRTI

In this section, several tests are implemented to evaluate the performance of MTRTI. The entropy theory, proposed by Shannon, is able to quantify the uncertainties of text, and the uncertainty is an important factor to evaluate the structure degree of the text. In the experiment, we examine the uncertainty of sample text before and after using MTRTI. Thus, if the data structuring process can obviously reduce the information uncertainty, the structuring method is workable. 

The evaluation process is divided into 3 phases, which are Sentence Splitting Phase (Phase I), Part-of-speech Tagging Phase (Phase II) and Temporal Information Extraction Phase (Phase III). According to theses phases, we developed the evaluation method flowingly.

Before Phase I, by using information entropy, we need to calculate the information entropy of this raw medical text. Since this medical text is just the raw text from the medical records without any extra information, the calculation of its entropy will be easy because it follows the basic equation of information entropy as in [20]:

(5) $$H_{n}=-\sum_{i=1}^{n}p_{i}\log_{2}p_{i}$$

In Equation (5), *H_n_* stands for the entropy of medical text, and in each of above phases, it should be re-calculated because of the different forms of the texts after the structuring process. *p_i_* stands for the occurrence probability of the single word shown in the texts. *n* stands for the total number of words in the texts. Since it does not contain extra information to describe the text, the probability of each word is the same (in Chinese, the total number of Chinese characters is set to 2500), so the maximum of information entropy of the specific texts can be calculated as *H_n_* = −log_2_*n*.

In Phase I, the information entropy of the texts can be calculated by Equation (6):

(6) $$H_{n}=\frac{1}{m}\sum_{i=1}^{m}\left(-\sum_{j=1}^{k_{i}}p_{i,j}\log_{2}p_{i,j}n_{j}\right)$$

where *m* stands for the number of the sentences in the text. *n_j_* stands for the number of words in the *j^th^* segment of the *i^th^* sentence. *p_i,j_* is the probability of the *j^th^* word of *i^th^* sentence. The entropy of text in this phase is the sum of the entropy of each sentence. In our model, a sentence contains many segments, and each of the segments belongs to a sentence. After the structuring process in this phrase, some grammatical relationships can be extracted, so the probability to identify those texts will depend on the position of segment in the sentence. *p_i,j_* still equals *p_i_* in Equation (5).

The second phase is to recognize the part-of-speech of each segment. In this phase, many terms are tagged properly, making it clearer in the context of the method. Thus, the uncertainty of the texts is continuously reduced. Basically, the equation to calculate the entropy is the same form as that in (6). However, the entropy of their segments in (6) can be calculated by Equation (7):

(7) $$H_{s}=-\sum_{i=1}^{c}p{\left(w\right)}_{i}\log_{2}p{\left(w\right)}_{i}$$

where *p*(*w*)*_i_* can be obtained by Equation (8):

(8) $$p{\left(w\right)}_{i}=-n_{w}p_{i}$$

in which *n_w_* is the length of the term.

Since we know the part-of-speech of the terms in this phrase, the probability of each term in Equation (7) can be seen as the degree of importance of a part-of-speech for the texts. Generally, nouns are much more important than prepositions. Thus, the probability of a noun is larger than that of preposition words. 

The last phase is to establish the temporal information model based on the structured texts in the second phase. In this phase, we reorganize the structured texts above by introducing the temporal information. By recognizing the part-of-speech of temporal information, we increase the probability of that temporal information in the texts, and recalculate their entropy of the texts as in:

(9) $$H_{tl}=\sum_{t=1}^{c}p_{t}H_{s}(t)$$

In the above equation, *H_tl_* is the entropy of proposed model involved with temporal information. *H_s_*(*t*) means the entropy of texts at *t^th^* time node, and *p_t_* stands for the probability for the medical researchers to find out the knowledge in the texts. 

**Figure 4:**
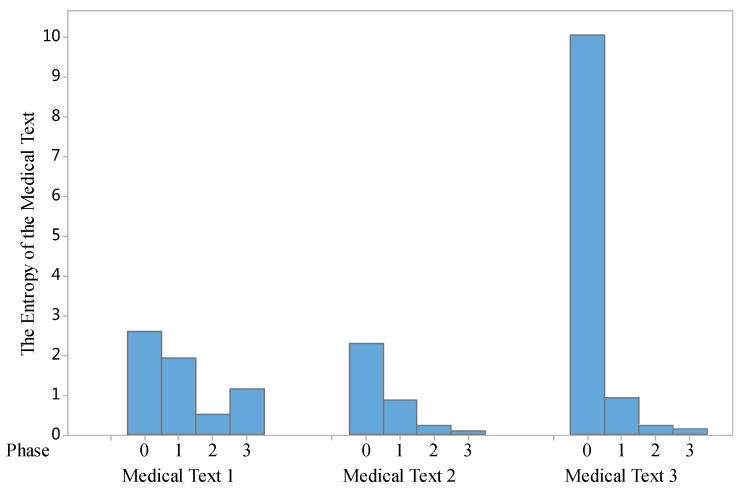
The changes of entropy in four phases (three selected EMR text samples).

Based on Equations (5)–(9), we implemented some entropy evaluation tests. The first test is to examine the changes of entropy in the three phases above, the evaluation results are shown in Figure 4.

As shown in Figure 4, by comparing the entropy of the specific medical texts (we select three samples of Chinese medical texts in three phases of the proposed model, it can be seen that the entropy of those medical free texts is reduced. This means the medical texts in Phase III (Temporal Information Extraction Phase) contains less uncertainty in illustrating the knowledge of the disease for patients. 

**Figure 5:**
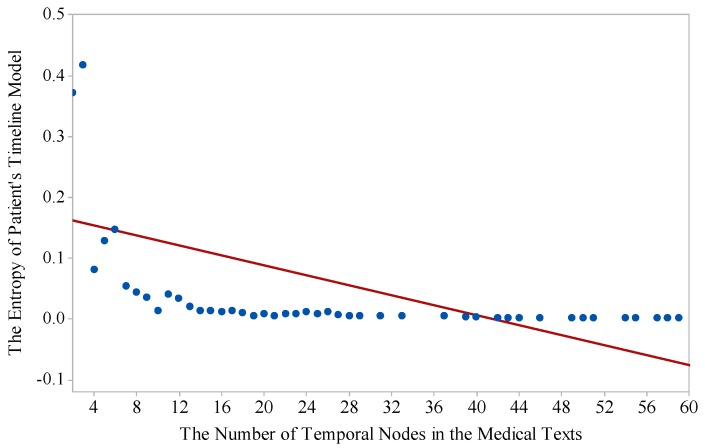
The changes of entropy with the increasing number of temporal nodes in EMR text.

The second test is to find out the relation between the entropy of the proposed model and the number of temporal nodes in the medical texts. The evaluation results are shown in Figure 5. In Figure 5, when the amount of temporal information increases, its entropy decreases. This indicates that more temporal information reduces the uncertainty of medical texts for the researchers, which proves that the temporal information is significant for knowledge extraction from medical texts. 

**Figure 6:**
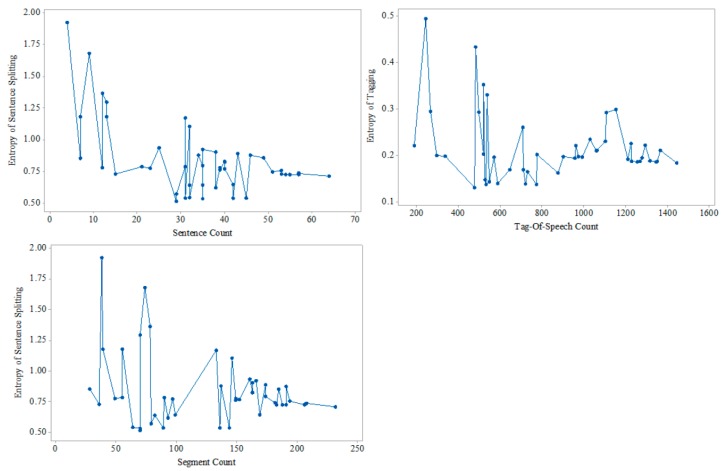
The changes of entropy with the increasing of the number of sentences, the number of part-of-speeches, and the number of segments.

At the same time, we analyze the influence of the number of sentences, the number of part-of-speech and the number of segments in the medical texts on the entropy of the proposed model. This is shown in Figure 6, where the increasing number of pieces of medical text agrees with the trend of entropy’s reduction. 

Entropy theory provided us with a powerful tool to compare the changes of information uncertainty in the text, and uncertainty is an important indicator to evaluate the text structural degree. According the information based on Equations (5)–(9) and Figure 4, Figure 5 and Figure 6, we are able to conclude that MTRTI reduces the uncertainty of texts in the medical texts for electronic medical records. In practice, it can help the medical researchers work on the computer-based structural data analysis method to mine the potential medical knowledge hidden in the free texts more easily.

## 5. Conclusions

In order to solve the problem of the difficulty for medical researchers in using medical free texts from accumulated Chinese electronic medical records for better medical diagnosis and treatment, this paper proposed a text structuring framework which is named as MTRTI based on the temporal information. MTRTI is a combination of several modules and models. MTRTI is effective at processing the unstructured EMR texts with temporal information into structured data. The experiments and tests compared the changes of complexity and the entropy when using MTRTI; as a result, it is indicated that the proposed framework is effective at reducing the information uncertainty. This work provide the possibility for the researchers who focus on structured medical data analysis, and enables computer-based medical analysis on the EMR text.

However, according to the evaluation on MTRII, there are also some shortcomings that need to be considered in the future. These texts only have little temporal information, so the entropy is still larger than that in the Phrase II (e.g., Medical Text 1 in Figure 4). This indicates that more temporal information in the texts will make it easier to implement the knowledge discovery procedure in the future. Another problem is in Equation (9). We set the same probability for each tagged word. In fact, different speech tags may have different usability for us. For example, a noun may be more important that a preposition. Thus, the entropy of texts will be less if we consider the different probability of different parts of speech.
